# Supplementary material for: Splice-Junction-Based Mapping of Alternative Isoforms in the Human Proteome
Source: Cell Rep. Author manuscript; Available in PMC 2020 Jan 15. (PMC6961840; doi:10.1016/j.celrep.2019.11.026)
Supplement: 2 [file NIHMS1546469-supplement-2.zip › DF1/PXD006675/AtrialSeptum/AtrialSeptum_25_CDV3_VQAMQISEK.pdf]

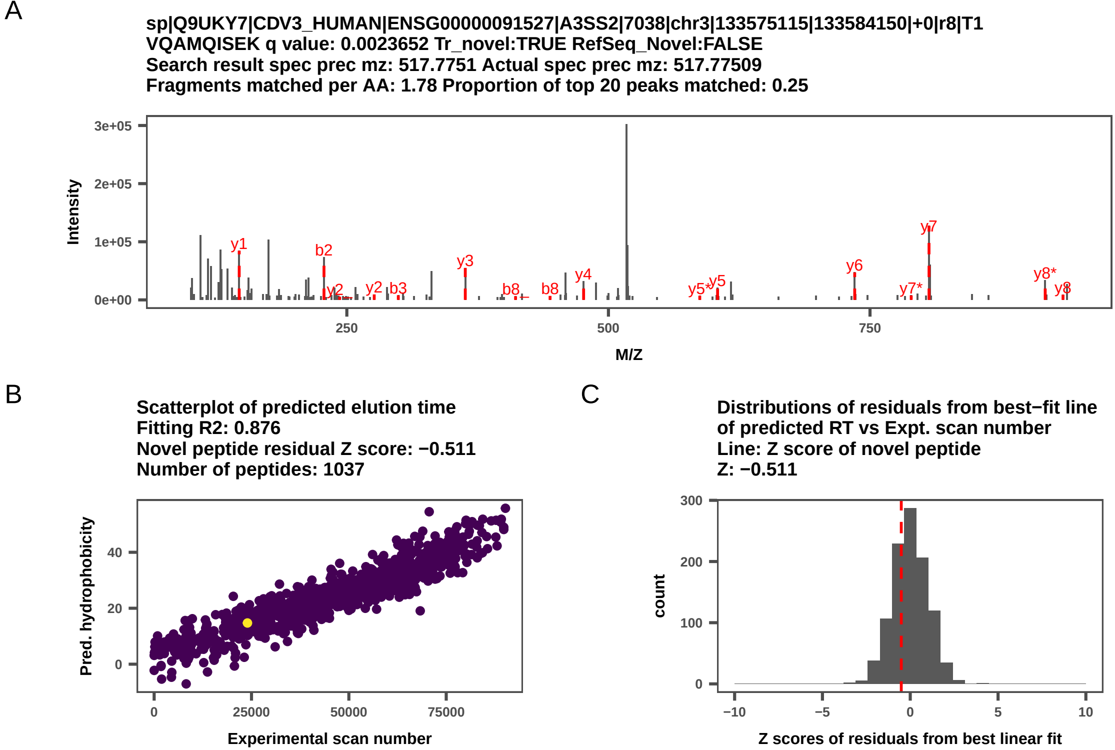
A
sp|Q9UKY7|CDV3_HUMAN|ENSG00000091527|A3SS2|7038|chr3|133575115|133584150|+0|r8|T1
VQAMQISEK q value: 0.0023652 Tr_novel:TRUE RefSeq_Novel:FALSE
Search result spec prec mz: 517.7751 Actual spec prec mz: 517.77509
Fragments matched per AA: 1.78 Proportion of top 20 peaks matched: 0.25
Intensity
3e+05
2e+05
1e+05
0e+00
y1
b2
y2
y2
b3
y3
b8
b8
y4
y5*
y5
y6
y7
y7*
y8*
y8
250
500
750
M/Z
B
Scatterplot of predicted elution time
Fitting R2: 0.876
Novel peptide residual Z score: −0.511
Number of peptides: 1037
Pred. hydrophobicity
40
20
0
0
25000
50000
75000
Experimental scan number
C
Distributions of residuals from best−fit line
of predicted RT vs Expt. scan number
Line: Z score of novel peptide
Z: −0.511
count
300
200
100
0
−10
−5
0
5
10
Z scores of residuals from best linear fit
